# Supplementary material for: Do human screams permit individual recognition?
Source: PeerJ. 2019 Jun 24;7:e7087. doi: 10.7717/peerj.7087 (PMC6596410; doi:10.7717/peerj.7087)
Supplement: Supplemental Information 2 — Ten-item survey developed for scream studies in our lab, with questions designed to assess exposure to screams in the media and confidence in making judgments during the experiment. [file peerj-07-7087-s002.pdf]

Participant number: ______________ Date: __________________

Male / Female Age: __________ Native (First) Language : __________________

---

1. I watch movies (movie theater, internet, TV, etc.):

1<br>Not often	2	3	4	5<br>Often

2. I enjoy scary movies:

1<br>Not at all	2	3	4	5<br>A lot

3. I watch scary or horror movies:

1<br>Not often	2	3	4	5<br>Often

4. I watch TV shows where screaming often occurs:

1<br>Not often	2	3	4	5<br>A lot

5. I consider myself knowledgeable about movies, the film industry, and accomplished actors.

1<br>Not at all	2	3	4	5<br>A lot

6. I consider myself knowledgeable about video games.

1<br>Not at all	2	3	4	5<br>A lot

7. I play action video games:

1<br>Not often	2	3	4	5<br>A lot

CONTINUED ON OTHER SIDE ....

8. I felt reasonably confident in my assessment of the screams I heard in this experiment.

1	2	3	4	5
Not confident				Very confident

9. How hard was it for you to make judgments about the different screams you heard in this experiment?

1	2	3	4	5
Very hard				Not hard

10. I consider myself quite good at reading emotions in people.

1	2	3	4	5
Not good				Very good

11. I am:

RIGHT HANDED   LEFT HANDED   AMBIDEXTROUS
