# Supplementary material for: Do human screams permit individual recognition?
Source: PeerJ. 2019 Jun 24;7:e7087. doi: 10.7717/peerj.7087 (PMC6596410; doi:10.7717/peerj.7087)
Supplement: Supplemental Information 3 — Some trials were omitted from analyses in the primary article because they either comprised duplicate stimulus pairs or reflected premature responses (i.e., responses provided after the second stimulus had started playing but before it had finished). Analyses were re-conducted including these trials, and the few results differing from those reported in the article (related to the effects of participant attributes) are noted here. [file peerj-07-7087-s003.docx]

**Discrepant results.** As noted in the primary article, some trials were omitted from analyses because they either (a) comprised duplicate stimulus pairs or (b) reflected premature responses (i.e., responses provided after the second stimulus had started playing but before it had finished). However, we re-analyzed the data in every possible combination (i.e., using different duplicate stimulus pairs and/or including premature responses). These alternate analyses were all consistent with those reported in the primary article and do not change our basic conclusions. However, the following results concerning participant attributes differed from those reported in the article:

- The small main effect of vocalizer gender on *d’* scores was not significant when premature responses were included (*p* = .077), nor when the stimulus duplicates noted in the Methods were used in the analyses (*p* = .146).
- In the analysis omitting Same-Duration Modified trials, a marginally significant effect of listener gender was found when alternate stimulus duplicates were analyzed (*p* = .047).
- The regressions of *d’* on participant attributes were marginally significant when premature trials were included in analyses, both including and excluding Same-Duration Modified trials. Those results are presented in the table below.

| *Multiple Linear Regression Results* | | | | | | | |
| --- | --- | --- | --- | --- | --- | --- | --- |
| Model | *t* | *p* | β | *F* | *df* | *p* | *R*^2^ |
| Including Same-Duration Modified trials |  |  |  | 2.41 | 5, 98 | 0.042 | 0.11 |
| Intercept | 5.850 | < .001 | 1.598 |  |  |  |  |
| F1. Media | 1.239 | .218 | .068 |  |  |  |  |
| F2. Video Games | 1.849 | .067 | .109 |  |  |  |  |
| F3. Confidence | 2.314 | **.023** | .129 |  |  |  |  |
| F4. Movies | .952 | .344 | .053 |  |  |  |  |
| EQ | .179 | .858 | .001 |  |  |  |  |
| Excluding Same-Duration Modified trials |  |  |  |  |  |  |  |
| Intercept | 4.605 | < .001 | 1.275 | 2.48 | 5, 98 | 0.037 | 0.11 |
| F1. Media | .984 | .328 | .055 |  |  |  |  |
| F2. Video Games | 2.177 | **.032** | .130 |  |  |  |  |
| F3. Confidence | 2.231 | **.028** | .126 |  |  |  |  |
| F4. Movies | .819 | .415 | .046 |  |  |  |  |
| EQ | .421 | .675 | .002 |  |  |  |  |

*Note.* Significant *p*-values (*p* < .05) are bolded.
